# Fear, distress, and perceived risk shape stigma toward Ebola survivors: a prospective longitudinal study

**DOI:** 10.1186/s12889-021-12146-0

**Published:** 2021-11-11

**Authors:** Cara M. Antonaccio, Phuong Pham, Patrick Vinck, Katharine Collet, Robert T. Brennan, Theresa S. Betancourt

**Affiliations:** 1 Research Program on Children and Adversity, Boston College School of Social Work, Boston, MA USA; 2 Harvard Medical School and Harvard T.H. Chan School of Public Health, Boston, MA USA; 3 Imperial College London School of Public Health, London, UK; 4 Women’s Studies Research Center, Brandeis University, Waltham, MA USA

**Keywords:** Infectious disease, Stigma, Ebola virus, Epidemic, Sierra Leone, Public health, Mental health, Global health, Neglected tropical diseases

## Abstract

**Background:**

During the 2014–15 Ebola Virus Disease (EVD) epidemic, thousands of people in Sierra Leone were infected with the devastating virus and survived. Years after the epidemic was declared over, stigma toward EVD survivors and others affected by the virus is still a major concern, but little is known about the factors that influence stigma toward survivors. This study examines how key personal and ecological factors predicted EVD-related stigma at the height of the 2014–2015 epidemic in Sierra Leone, and the personal and ecological factors that shaped changes in stigma over time.

**Methods:**

Using three waves of survey data from a representative sample in the Western Urban and Western Rural districts of Sierra Leone, this study examines factors associated with self-reported personal stigma toward Ebola survivors (11 items, α = 0.77) among 1008 adults (74.6% retention rate) from 63 census enumeration areas of the Western Rural and Western Urban districts of Sierra Leone. Participants were randomly sampled at the height of the EVD epidemic and followed up as the epidemic was waning and once the epidemic had been declared over by the WHO. Three-level mixed effects models were fit using Stata 16 SE to examine cross-sectional associations as well as predictors of longitudinal changes in stigma toward EVD survivors.

**Results:**

At the height of the EVD epidemic, female sex, household wealth, post-traumatic stress, EVD-related fear and perceived infection risk are a few of the factors which predicted higher levels of stigma toward survivors. On average, stigma toward EVD survivors decreased significantly as the epidemic declined in Sierra Leone, but female sex, EVD fear, and risk perceptions predicted a slower rate of change.

**Conclusion:**

This study identified key individual and psychosocial characteristics which may predict higher levels of stigma toward infectious disease survivors. Future studies should pursue a better understanding of how personal characteristics and perceptions, including psychosocial distress, fear, and perceived infection risk serve as pathways for stigma in communities affected by infectious disease.

**Supplementary Information:**

The online version contains supplementary material available at 10.1186/s12889-021-12146-0.

The 2014–2015 Ebola virus epidemic in Sierra Leone resulted in thousands of people becoming infected with the virus and left tens of thousands of families broadly affected. In Sierra Leone, Ebola virus disease (EVD) affected an estimated 14,122 confirmed, probable and suspected people, among whom 3955 died; however, thousands more survived after a confirmed EVD diagnosis [1]. Long before the EVD outbreak, Sierra Leone’s 11-year civil war (1991–2002) resulted in the deaths of over 50,000 people, devastating the country’s health system and fracturing its social fabric. As a result, Sierra Leone’s health system was extremely vulnerable to the EVD outbreak. Resulting from the failure to strengthen and sustain health and social systems after the war, many survivors of EVD are faced with healthcare neglect, discrimination, and social exclusion [2, 3].

To date, little research has been done to examine the nature, predictors, and effects of stigma toward survivors of infectious disease outbreaks like the 2014–15 EVD epidemic in West Africa. Although some studies such as James et al. (2019) and (2020) have investigated the experiences of EVD survivors, themselves, an enduring gap in the literature persists regarding the key factors that predict stigma and discrimination against people affected by EVD and how, if at all, stigma is affected by trends in infection rates. Despite the persistent gap in EVD-related evidence, research on stigma among individuals affected by HIV/AIDS is informative in the study of stigma toward survivors of EVD. For example, people living with HIV/AIDS who experience stigma and discrimination report high rates of comorbid mental health problems, poor adherence to treatment, among other harmful effects [4].

Research on HIV/AIDS also underscores factors which may predict stigma toward those affected by EVD and other infectious diseases like COVID-19. For example, evidence from HIV/AIDS research suggests that the perceived contagiousness and degree to which physical problems are manifest appear to influence stigma and discrimination against people with the disease [5]. Similarly, research in the context of HIV/AIDS points to several personal and ecological factors thought to contribute to infectious disease-related stigma. Lessons from HIV/AIDS research offer a strong foundation for stigma research and planning in response to the 2014–15 EVD outbreak in West Africa and 2019–2020 EVD outbreaks in Democratic Republic of Congo and Guinea. The recent (2021) EVD outbreak in Guinea is especially concerning, as it underscores the real possibility that new EVD outbreaks may emerge in Sierra Leone and elsewhere. Overall, more evidence is needed about key predictors of stigma and discrimination, as this informative is important in design of evidence-based approaches to prevent stigma toward survivors of other infectious diseases, including the ongoing global COVID-19 pandemic.

This study uses a prospective longitudinal design to examine predictors and trends of stigma toward EVD survivors, cross-sectional associations, and changes in stigma from the height of the 2014–2015 EVD outbreak in Sierra Leone until after it was declared over. First, we explore which individual, social, ecological factors, if any, are associated with stigma toward EVD survivors in their community at the height of the outbreak; second, we investigate how, if at all, stigma toward EVD survivors in Sierra Leone changed as the epidemic declined; and third, we examine how, if at all, individual, social, and ecological factors predicted changes in stigma toward adult EVD survivors from the height of the 2014–2015 until it was declared over.

## Methods

**Fig. 1:**
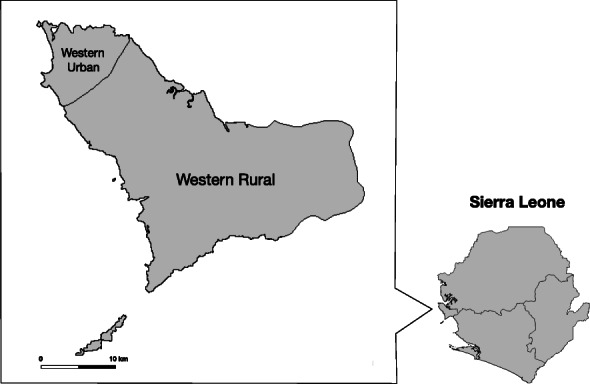
Map by the authors, based on 2014 Sierra Leone administrative areas in GADM, version 2.8 (http://www.gadm.org)

Surveys were conducted in the Western Rural and Western Urban districts of Sierra Leone (including the capital, Freetown) at three time points: January to April 2015, March to September 2015, and February to April 2016 (Fig. 1). During the 2014–15 outbreak, this region of Sierra Leone was among the worst affected by EVD, where over 40% of confirmed cases occurred. Census enumeration areas (EAs) were the primary sampling units. To facilitate sampling, Statistics Sierra Leone provided a list of EAs for the two districts and maps defining the EA boundaries as well as a map with specified number of streets (2–5) among each of the EAs following procedures for the Sierra Leone 2004 Population and Housing Census [6].

Ethical review and approval for this study were obtained from the Sierra Leone Ministry of Health and Sanitation Ethics and Scientific Review Committee as well as the Institutional Review Board of the Harvard T. H. Chan School of Public Health (Protocol #15145, Approval #17). All participants in the survey were over the age of 18 and enrolled following informed consent delivered by a team of 16 trained local staff. All participants gave informed consent orally due to low levels of literacy in the sample. All research methods and protocols were reviewed and approved by a local community advisory board comprising community members and health care professionals.

### Sample

**Fig. 2:**
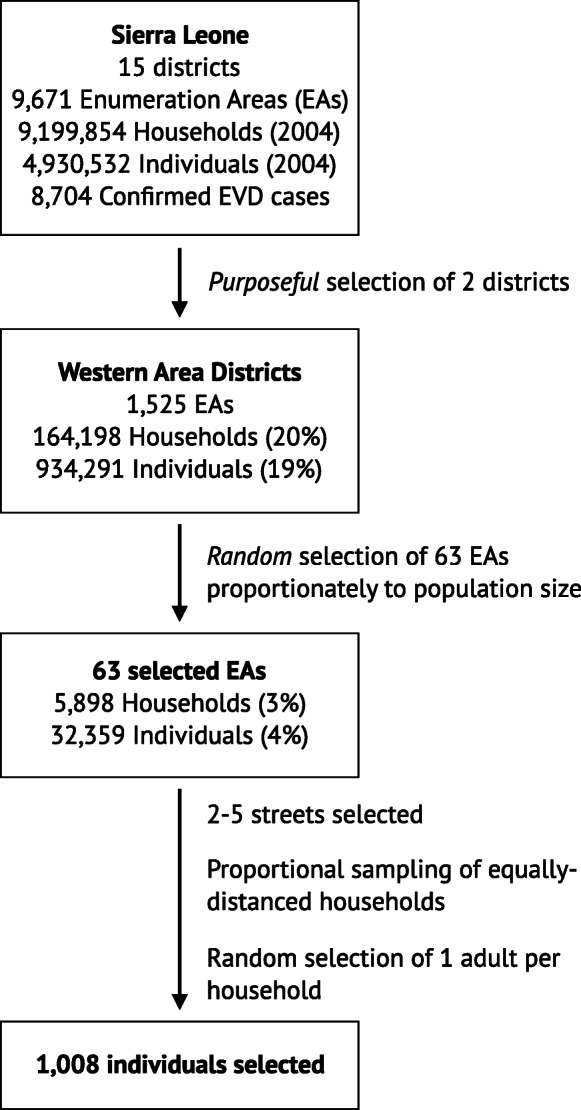
Sampling procedure for the study

Participants in the study were over 18 years of age and sampled using multistage cluster sampling in the Western Urban and Western Rural districts of Sierra Leone (Fig. 2). Of the 9671 enumeration areas (EAs) in the Western Area Urban and Western Area Rural districts of Sierra Leone, 63 EAs were randomly sampled. Within each EA, 16 households were selected using random geographic sampling techniques. Interviewers then selected a proportional sample of equally distanced households (“households” were defined as persons residing together). Households were approached and one adult among those available on first contact was chosen at random from the household by alphabetizing first names in ascending order and choosing the first one. When a randomly selected individual was unable or refused to participate, another individual from the same household was selected using the randomization procedure. Over the course of 1 day, if no member was available after three attempts, another household was selected using the same geographic randomization techniques. Similarly, if a participant who was interviewed at the first wave of data collection could not be located at the second or third wave following three attempts, another eligible adult in the household was interviewed.

### Procedures

A team of 16 local research assistants (eight women and eight men) were trained in survey administration using Android tablets running Kobo Toolbox digital data collection software [7]. All research assistants received 3 days of training in survey procedures and research ethics and interviewers were assigned to interview participants of the same sex. Despite the challenging work conditions during the ongoing EVD epidemic, the study team and Community Advisory Board (CAB) determined that one-on-one interviews could be performed safely in an outdoor location close to participants’ homes that provided privacy and confidentiality. Oftentimes, this private location was a compound away from family members and neighbors, or a nearby sitting area. To ensure the safety of respondents and enumerators, precautions were taken, such as no physical contact and sitting at a safe social distance to complete interviews. Participants were given $2 worth of food for their participation, as well as contact information in case they had any questions after participating in the study. In the event of a risk of harm situation, outreach was conducted with local medical and social service providers. Two such cases, related to suicidal ideation, were identified and referred to local mental health and social services for follow-up care.

### Measurement

All measures that were new to this setting were reviewed by a local community advisory board (CAB) and also by local collaborators for face validity. Scales were examined for local comprehension and forward- and backward-translated following a standard protocol [8–10]. Stigma toward EVD survivors (Cronbach’s α = 0.77) was measured using a Krio (the lingua franca of Sierra Leone) translation of an eleven-item adaptation of the HIV-related stigma scale, with items scored as a 4-point Likert scale. Items on the scale assessed whether a respondent agreed (1 = strongly disagree, 2 = somewhat disagree, 3 = somewhat agree, 4 = strongly agree) with statements designed to assess stigma and discriminatory attitudes toward individuals who were given a certificate to confirm their EVD-free status by the government health authority (Table S1). To assess personal EVD exposure, participants were asked whether a member of their household, a family member whom they did not live with, a friend, a neighbor, or someone in their community had been diagnosed with EVD in the past 12 months (0 = no, 1 = yes). To examine the effect of anti-stigma messaging, one item asked respondents whether they had seen or heard any messages, in the past month, condemning discrimination and stigmatization of Ebola survivors (0 = no, 1 = yes).

Scales previously validated for use in Sierra Leone, Liberia, and other conflict-affected countries in sub-Saharan Africa were used to assess mental health [11–16]. Depression and anxiety symptoms were assessed using a Krio adaptation of the Hopkins Symptom Checklist-25 (HSCL-25), scored on past week symptom intensity (1 = not at all, 2 = a little, 3 = quite a bit, 4 = extremely), previously adapted for Sierra Leone [11–13]. Both the depression (Cronbach’s α = 0.91) and anxiety (Cronbach’s α = 0.93) subscales had excellent internal consistency. PTSD symptoms were assessed using a 16-item scale (0 = no, 1 = yes), the PTSD Symptom Scale–Interview, an adaptation of the Civilian PTSD checklist used in Liberia (Cronbach’s α = 0.93) [14]. Perceived risk of EVD infection was measured using three items that assessed whether participants were concerned that they themselves, someone in their family, and someone in their community would get sick with Ebola in the following month (Cronbach’s α = 0.96).

Ebola-related fear was measured using ten Likert-style questions about whether certain daily activities made them fearful in the context of the ongoing EVD outbreak (α = 0.76). To capture health care-seeking behaviors and contact with health formal and informal health services, respondents were asked about the how many times in the previous 12 months they sought treatment for health concerns from traditional healers and at a hospital or clinic, respectively. Last, to examine participants’ EVD-related social ecology, two scales we used to assess respondent perceptions of leadership efficacy and community resilience. In scoring, the means of all scales were used to facilitate interpretation in terms of their original response scales. Limitations of the study design include the potential for bias arising from the repeated survey designs, including respondent bias and social desirability bias.

### Analysis

We used Stata 16 SE to conduct descriptive and inferential statistical analyses [17]. To better understand predictors of stigma toward EVD survivors, we estimated three-level hierarchical linear models (also called “multilevel” or “mixed” effects models) to accommodate the clustering of participant data within EAs and over time and to reduce consequent biases in the estimation of standard errors. For model interpretation, we used robust standard errors. Because the study used an equal allocation sampling design with 16 households per EA, we applied sampling weights based on the population of each EA to allow for generalization to the Western Urban and Western Rural districts of Sierra Leone where EAs were sampled from. Upon review of the data, we determined that households in two EAs were undersampled due to a clerical error and up-weighted the remaining households so that those EAs would be properly represented in the sample. We placed misidentified households in their proper EAs, resulting in oversampling of those EAs. In analysis, households in these EAs were down-weighted to prevent overrepresentation in the weighted sample.

To study basic trends in stigmatizing attitudes and behaviors, we observed longitudinal changes in participants’ stigma toward EVD survivors, from the height of the outbreak until it was declared over. We fit mixed effects models in Stata 16 SE to examine predictors of stigma and longitudinal growth. To retain and use all available data and avoid possible bias associated with listwise missing value deletion, we created 20 multiply imputed datasets for the analyses, using all analysis variables plus additional demographic measures (participant age, sex, marital status, education, and household wealth, based on household land and asset ownership). The number of missing values for a given variable can be determined by comparing the *n* for a given variable in Tables 1 and 2 (e.g., 979 for depression symptoms score) to 1008, the total number of participants interviewed.

**Table 1:** Demographic, social, and economic characteristics of respondents

Characteristic	N	Unweighted Percent (95% CI)	Weighted Percent (95% CI)
**Sex**			
Male	503	49.9	49.7
Female	505	50.1	50.2
**Age** (mean, years)	1008	33.8 (32.9, 34.6)	4.2 (33.2, 35.2)
**Household assets** (mean, of 9)	1008	3.6 (3.5, 3.8)	3.5 (3.2, 3.9)
**Marital status**			
Currently married	424	42.1 (39.0, 45.1)	42.7 (38.8,46.7)
Widowed	49	4.9 (3.7, 6.4)	6.0(4.4, 8.1)
Cohabiting but not married	21	2.1 (1.4, 3.2)	2.1 (1.2, 3.5)
Single/Never married	455	45.1 (42.1, 48.2)	43.2 (39.4, 47.1)
Other^a^	59	5.9 (4.6, 7.5)	6.0 (4.5, 8.1)
**Education**			
No formal education	163	16.2 (14.0, 18.6)	18.5 (14.6, 23.2)
Some primary	48	4.8 (3.6, 6.3)	4.7 (3.5, 6.3)
Completed primary	17	1.7 (1.1, 2.7)	1.7 (0.9, 3.1)
Some secondary	380	37.8 (34.8, 40.8)	37.9 (34.0, 41.9)
Completed secondary	239	23.8 (21.2, 26.5)	22.0 (18.7, 25.6)
Some post-secondary	86	8.5 (7.0, 10.4)	8.2 (6.5, 10.4)
Completed post-secondary	73	7.3 (5.8, 9.0)	7.0 (5.5, 9.0)
**Ethnicity**			
Temne	368	36.5 (33.6, 39.5)	40.1 (34.3, 46.1)
Mende	161	16.0 (13.8, 18.4)	15.6 (12.7, 19.2)
Limba	134	13.3 (11.3, 15.5)	13.6 (10.6, 17.2)
Krio	77	7.6 (6.1, 9.5)	6.6 (4.9, 8.9)
Fulah	74	7.3 (5.9, 9.1)	6.3 (4.6, 8.6)
Loko	45	4.5 (3.3, 5.9)	4.3 (3.2, 5.9)
Mandingo	45	4.5 (3.3, 5.9)	4.3 (3.0, 6.2)
Soso	31	3.1 (2.2, 4.3)	2.9 (1.9, 4.2)
Kono	20	2.0(1.3, 3.1)	1.8 (0.9, 3.3)
Other^b^	53	5.3 (4.0, 6.8)	4.5 (3.0, 6.7)
**Language**			
Krio	882	87.5 (85.3, 89.4)	88.4 (85.3, 91.0)
Temne	64	6.3 (5.0, 8.0)	5.8 (4.0, 8.3)
Mende	20	2.0 (1.3, 3.1)	1.7 (1.1, 2.8)
Fulah	17	1.7 (1.1, 2.7)	1.8 (1.1, 2.9)
Limba	10	1.0 (0.5, 1.8)	1.2 (0.6, 2.5)
Other^c^	15	1.5 (0.9, 2.5)	1.1 (0.4, 2.5)
**Household owns land**			
No	801	79.6 (77.0, 82.0)	77.9 (73.3, 81.8)
Yes	205	20.4 (18.0, 23.0)	22.1 (18.2, 26.7)

Survey results are representative of the adult household-based population of the Western Urban and Western Rural districts of Sierra Leone at each of the three waves of data collection, between January 2015 and April 2016.

^a^ 15 respondents lived with a partner but were now single, 14 were engaged, nine were separated but married, seven were divorced, and 14 other

^b^ Eight Kissi, five Valunka, two Kru, one Marankais, and 37 other

^c^ Two Kono, one English, and 12 other

**Table 2:** Estimated mental health and personal EVD exposure characteristics

Characteristics (***n =*** 1008) ^a^	Time 1		Time 2		Time 3	
	Unweighted Percent (95% CI)	Weighted Percent (95% CI)	Unweighted Percent (95% CI)	WeightedPercent (95% CI)	Unweighted Percent (95% CI)	Weighted Percent (95% CI)
**Mental health**						
Depression score, mean	1.38 (1.35, 1.42)	1.39 (1.30, 1.48)	1.40 1.37, 1.43)	1.43 (1.34, 1.51)	1.31 (1.27, 1.33)	1.35 (1.27, 1.42)
Anxiety score, mean	1.29 (1.26, 1.33)	1.29 (1.21, 1.38)	1.31 (1.28, 1.34)	1.31 (1.22, 1.40)	1.18 (1.17, 1.20)	1.20 (1.17, 1.22)
Posttraumatic stress symptom score, mean	0.18 (0.16, 0.21)	0.20 (0.15, 0.24)	0.25 (0.22,0.27)	0.26 (0.20, 0.33)	0.15 (0.13, 0.17)	0.18 (0.12, 0.23)
**Personal exposures to EVD**						
Someone in the community (*n* = 1001)	41.5 (38.40, 44.60)	44.4 (34.60, 54.28)	16.1 (13.7, 18.43)	18.4 (12.44, 24.31)	12.6 (10.3, 14.98)	15.2 (7.0, 23.52)
Neighbor (*n* = 1003)	17.5 (15.20, 19.93)	19.8 (13.14, 26.59)	12.8 (10.62, 14.89)	14.5 (8.96, 20.11)	9.0 (6.98, 11.01)	11.1 (4.70, 17.68)
Friend (*n* = 1002)	9.2 (7.44, 11.05)	10.3 (5.78, 14.83)	6.2 (4.62, 7.70)	6.9 (3.93, 9.77)	1.7 (0.77, 2.60)	2.1 (2.57, 3.91)
Non-household family member (*n* = 1001)	8.7 (6.10, 10.51)	10.8 (6.02 15.5)	5.9 (4.35, 7.36)	6.1 (3.69, 8.49)	5.46 (3.85, 7.07)	6.2 (1.89, 10.58)
Household member (*n* = 1001)	5.3 (3.93, 6.73)	6.6 (2.69, 10.45)	2.4 (1.45, 3.43)	3.0 (1.65, 4.27)	1.9 (0.95, 2.88)	2.0 (0.51, 3.50)

^a^ Survey results are representative of the adult household-based population of the Western Urban and Western Rural districts of Sierra Leone from April 2015 to April 2016. Equal allocation sampling design was based on 16 households per EA, sampling weights were based on the population of each EA to allow generalization to the Western Urban and Western Rural districts of Sierra Leone from which the EAs were sampled

### Hierarchical modeling approach

Our statistical modeling approach was hierarchical such that we added predictors to the unconditional growth model in 7 groups, retaining all predictors notwithstanding statistical significance in previous models. We compared each subsequent model to the null growth model as well as the preceding model to examine the proportion of variance in stigma explained by the added predictors. We specified all models with unstructured covariance to prevent Stata from setting the covariance and corresponding correlations to zero by default. We estimated deviance values to determine the fit of each model. Stata’s mixed program uses a Bayesian approach to estimate the hierarchical linear model and calculates the mean of the a posteriori distribution of the random effect with robust standard errors. With respect to the model properties, Rabe-Hesketh and Skrondal (2012) explain that Stata mixed model results are conditionally biased, such that for any individual cluster the estimation is biased; however, the advantage of Stata’s estimation method is that the conditional bias is countered by a lower mean-squared error for the entire population [18].

## Results

Table 1 presents the population-weighted social and demographic characteristics in the adult household-based sample from the Western Urban and Western Rural districts of Sierra Leone. The sample comprised 505 women (50.8%) and 503 men (49.2%) with a mean age of 34.2 years (95% CI: 33.2, 35.2) and median age of 30 years. Ages ranged from 18 to 84 years. Nearly half of the participants were married or partnered (42.7%; 95% CI: 38.8, 46.7%) and an approximately equal proportion were single or did not have a partner (45.1%; 95% CI: 42.1, 48.2%). About one-fifth of the participants reported having no formal education (18.5%; 95% CI: 14.6, 23.2%) or incomplete primary school (4.7%; 95% CI: 3.5, 6.3%). The main ethnic groups in the sample were Temne (40.1%; 95% CI: 34.3, 46.1%), Mende (15.6%; 95% CI: 12.7, 19.2%), and Limba (13.6%; 95% CI: 10.6, 17.2%). Most participants (88.4%; 95% CI: 85.3, 91.0%) spoke Krio at home. From a list of nine common household assets, participants owned a mean of 3.5 assets (95% CI, 3.2, 3.9). Table 2 reports the population-weighted estimates of mental health outcomes and EVD exposures at each timepoint. At the height of the epidemic, close to half of participants reported having EVD cases in their community (44.7%; 95% CI: 35.6, 54.3%), and one in five reported cases among neighbors (20.5%; 95% CI: 14.3, 28.4%). Fewer participants reported cases among friends (11.0%; 95% CI: 6.8, 17.3%), family members residing outside the household (11.1%; 95% CI: 7.2, 16.6%), and household members (6.9%; 95% CI: 3.8, 12.3%).

### Multilevel data structure

We estimated two intraclass correlations (ICC) for the three-level mixed effects model to assess the similarity of stigma scores between respondents from the same enumeration area and within individuals over time. The ICC at level-3 (the correlation of responses from individuals in the same EA) was equal to 0.11. The low level-3 ICC suggests that only a small amount of the variance in stigma responses is attributed to differences between individuals from the same enumeration area. The level-2 ICC also indicates a modest within-person correlation of 0.18, indicating that there was not a high correlation between stigma scores from the same respondent over time.

#### Longitudinal, hierarchical linear models predicting stigma toward EVD survivors

In the null model, the fixed effect for initial stigma status is the mean of the first plausible value for stigma in the sample, not accounting for between-enumeration area, between-person differences, or within-person variation over time. The first plausible stigma score in the null model is estimated as 0.586. In the unconditional growth model, the grand mean for stigma in the sample accounts for random between-enumeration area, between-person, and within-person variation. Accounting for the between-enumeration area, between-person, and within-person variance, the mean of stigma in the unconditional growth model was estimated as 0.582 – slightly lower than the mean in the same model without random effects. Controlling for all personal, social, and ecological factors, the estimated mean stigma score among respondents at the height of the outbreak was equal 0.44 (95% CI: 0.25, 0.64).

Based on the analysis of residuals comparing the full model with all variables and the null unconditional growth model, sociodemographic characteristics, EVD exposures, mental health, fear, and perceived risk to EVD infection explain 57% of the variance in stigma at the height of the epidemic. Sociodemographic characteristics (i.e., sex, age, education, marital status, and household wealth) explained 7% of the variance in stigma at the height of the outbreak. EVD-related exposures (i.e.. someone in the community, neighborhood, friend, non-household family, and household family) explained only 4% of the variance in stigma. Mental health symptoms, fear and risk perceptions explained 16 and 31% of the residual variance in stigma, respectively, at the height of the outbreak. Neither health care-seeking behaviors nor ecological trust contributed any additional information, with 0.0% variance explained by the variables in both models. Based on the sample-size adjusted-BIC, the full model retaining all individual and ecological predictors fits the data better (BIC = 944.85) than the unconditional growth model (BIC = 2969.98).

#### Predictors of stigma toward EVD survivors at the height of the 2014–15 epidemic

On average, several individual and ecological characteristics predicted significantly higher levels of stigma toward EVD survivors at the height of the outbreak (Table 3). Female respondents reported significantly higher levels of stigma compared to male respondents (Cohen’s d = 0.818; t = 7.04, *p* = 0.000) and respondents from wealthier households also reported higher levels of stigma toward EVD survivors (Cohen’s d = 0.082; t = 2.47, *p* = 0.014). Interestingly, respondents’ physical proximity to Ebola survivors living in their community was not significantly associated with stigma at the height of the epidemic; however, those who had been exposed to messaging intended to prevent EVD-related stigma reported higher levels of stigma toward EVD survivors compared to those who had not been exposed to anti-stigma messaging (Cohen’s d = 0.445; t = 0.14, *p* = 0.001).

**Table 3:** Estimated effect of predictors on stigma toward EVD survivors at the height of the 2014–15 outbreak

Variable	Coef.	Robust SE	***t***-statistic	95% CI
Intercept	0.445	0.10		0.2528, 0.6370
Age	0.002	0.01	0.68	−0.0041, 0.0087
Female	0.818	0.12	7.04	0.5902, 1.0451
Household wealth	0.082	0.03	2.47	0.0169, 0.1470
Some primary education	−0.054	0.05	−1.16	−0.1445, 0.0368
Completed primary education	−0.032	0.05	−0.6	− 0.1373, 0.0728
Some secondary education	0.034	0.03	1.02	−0.0332, 0.1003
Completed secondary education	0.022	0.03	0.64	−0.0460, 0.0903
Married or engaged	− 0.001	0.02	−0.04	− 0.0464, 0.0446
Cohabiting, not married	−0.047	0.04	−1.22	−0.1235, 0.0290
Widowed	0.020	0.05	0.38	−0.0848, 0.1251
Divorced	−0.087	0.08	−1.04	−0.2512, 0.0766
Someone in the community	−0.400	0.22	−1.84	−0.8247, 0.0249
Neighbor	0.397	0.24	1.66	−0.0730, 0.8667
Friend	−0.046	0.27	−0.17	−0.5656, 0.4740
Non-household family	0.120	0.21	0.60	−0.2890, 0.5455
Household member	0.375	0.34	1.17	−0.2508, 0.9998
Anti-stigma messaging	0.445	0.14	3.24	0.1756, 0.7146
Depression	0.273	0.18	1.51	−0.0811, 0.6262
Anxiety	0.325	0.18	1.76	−0.0376, 0.6871
PTSD	1.144	0.24	4.77	0.6742, 1.6137
EVD fear	0.971	0.16	6.08	0.6582, 1.2837
Perceived risk	0.178	0.05	3.41	0.0755, 0.2799
Health care-seeking (clinic)	0.009	0.01	−0.79	−0.0233, 0.0412
Health care-seeking (traditional)	0.003	0.02	0.18	−0.0350, 0.0418
Trust in local leaders	0.109	0.10	1.07	−0.0766, 0.0794
Community resilience	−0.100	0.16	−0.61	−0.1270, 0.1360

Post-traumatic stress symptoms (Cohen’s d = 1.14; t = 4.77, p = 0.000), EVD-related fear (Cohen’s d = 0.97; t = 6.08, *p* = 0.000), and perceived EVD risk (Cohen’s d = 0.178; t = 3.41, *p* = 0.001) were all strongly positively associated with stigma toward survivors at the height of the outbreak. Neither a recent history of health care-seeking at a local clinic nor with traditional healers was found to be significantly associated with stigma toward EVD survivors at the height of the epidemic. Leader trust and perceptions of community resilience had no significant effect on stigma. On average, there was a small but non-significant reduction in stigma toward EVD survivors from the height 2014–15 EVD epidemic in Sierra Leone until it was declared over (Cohen’s d = − 0.02; t = − 0.29, *p* = 0.711). Although stigma toward EVD survivors in the sample attenuated on average as the outbreak declined, certain personal characteristics and perceptions predicted a slower rate of change (Table 4). Female respondents (Cohen’s d = − 0.208; t = − 4.65, *p* = 0.000) and respondents with above-average household wealth (Cohen’s d = − 0.028; t = − 2.14, *p* = 0.032) demonstrated a slower rate of change in stigma toward EVD survivors, on average. Other personal characteristics that predicted a slower rate of change in stigma included post-traumatic stress (Cohen’s d = − 0.524; t = − 5.09, *p* = 0.000), EVD fear (Cohen’s d = − 0.379; t = − 5.81, *p* = 0.000) and perceived infection risk (Cohen’s d = − 0.054; t = − 2.50, *p* = 0.012). Interestingly, none of the predictors in the conditional growth models predicted a statistically significant increase in the rate of change in stigma toward EVD survivors.

**Table 4:** Predictors of longitudinal growth in stigma toward EVD survivors from April 2015 to April 2016

Characteristic	Cohen’s ***d***	Robust SE	***t***-statistic	95% CI
Age	−0.001	0.01	−0.52	− 0.0042, 0.0087
Female	−0.208	0.04	−4.65	0.5902, 1.0451
Household wealth	−0.028	0.01	−2.14	0.0169, 0.1470
Someone in the community	0.142	0.09	1.62	−0.0295, 0.3135
Neighbor	−0.170	0.10	−1.78	− 0.3573, 0.0168
Friend	0.017	0.12	0.15	− 0.2116, 0.2451
Non-household family	−0.059	0.09	−0.67	− 0.2308, 0.1130
Household member	− 0.134	0.13	−1.07	−0.3794, 0.1115
Anti-stigma messaging	−0.143	0.06	−2.50	−0.2556, − 0.0310
Depression	− 0.066	0.07	−0.90	− 0.2089, 0.0777
Anxiety	−0.054	0.08	−0.67	− 0.2118, 0.0138
PTSD	−0.524	0.10	−5.09	−0.7252, − 0.3219
EVD fear	− 0.379	0.07	− 5.81	− 0.5075, − 0.2514
Perceived risk	− 0.054	0.02	−2.50	0.0755, 0.2799
Health care-seeking (clinic)	−0.006	0.01	0.18	−0.0213, 0.0905
Health care-seeking (traditional)	−0.005	0.01	−0.55	−0.0211, 0.0119
Trust in local leaders	0.001	0.04	0.04	−0.0766, 0.0794
Community resilience	0.007	0.07	0.01	−0.1227, 0.1376

## Discussion

This study examined predictors of stigma toward survivors at the height of the 2014–15 EVD outbreak in Sierra Leone and factors that shaped change in stigma over time. On average, female participants demonstrated higher levels of stigma at the height of the outbreak compared to male participants, controlling for all other variables. Similarly, participants with above-average household wealth reported significantly higher levels of stigma toward EVD survivors in their community. Mental health and psychosocial factors, including post-traumatic stress, EVD-related fear and risk perceptions were also positively and significantly associated with stigma at the height of the outbreak. A significant decrease in stigma was observed from the height of the epidemic until it was declared over, but several individual and psychosocial factors predicted higher levels of stigma toward survivors of EVD. Female participants, as well as participants from wealthier households, demonstrated a slower rate of change in stigma toward EVD survivors. Participants with above average post-traumatic stress symptoms, EVD-related fear, and risk perceptions also demonstrated a slowed rate of change in stigma compared to those without these characteristics.

The finding that anti-stigma messaging was associated with elevated stigma was unexpected and warrants further investigation, as it could emphasize that the design and implementation of anti-stigma messaging are key to success. The uncontrolled spread of misinformation and falsehoods regarding EVD and the epidemic at the start of data collection may have been instrumental in increasing stigma and discrimination people who have had and who were affected by the infectious disease. In particular, at the start of the EVD outbreak in Sierra Leone, misinformation and unclear public health messaging gave rise to risk behaviors like secret burials and other potential harms linked with EVD-related fear and stigma. Misconceptions like these may have counteracted anti-stigma messaging, or been stigmatizing themselves. Similarly, throughout the study period, data collection was ongoing to understand EVD viral reservoirs within the body and routes of transmission. Rumors and misinformation about this work proliferated through the Western Areas and led many to believe that EVD could be sexually transmitted by survivors over a long period of time. Finally, it is possible that the observed relationship between anti-stigma messaging exposures and higher levels of stigma toward survivors may be an artefact of Sierra Leone’s reliance on government-issued certificates to prove EVD-free status.

## Conclusion

This study found that female sex, mental distress, EVD fear, and risk perceptions were all important predictors of stigma toward EVD survivors over the course of the 2014–15 epidemic in Sierra Leone. The findings offer important insights into stigma both EVD toward survivors as well as survivors of other infectious diseases. The findings also raise important questions about the role of health communication strategies and media outlets as major factors contributing to stigma and discrimination against infectious disease survivors. As the world continues to grapple with the effects of the ongoing COVID-19 pandemic, several similarities shared between the current SARS-COV-2 pandemic and recent EVD epidemics stand out; namely the high degree of contagiousness, proliferation of virus- and outbreak-related misinformation across media platforms, and targeting of certain social groups for blame related to the outbreak. In light of the growing importance of social and other forms of media as sources of health information, more research is needed to understand how the form, content, and delivery of health communication, broadly, and anti-stigma campaigns, in particular, contribute to the effectiveness of such approaches. In communities affected by infectious disease epidemics, approaches to reduce distress, fear, and the spread of misinformation may serve an important dual purpose -- preventing new infections as well as stigma and discrimination toward those affected by the disease.

## Supplementary Information

**Additional file 1.**

## Data Availability

The deidentified data used to reach the conclusions and replicate the analyses described in this manuscript are available upon written request to, and after review and approval by, the Harvard LMA Institutional Review Board (IRB), in order to protect participant privacy. The contact information for the IRB is: Leslie Howes, MPH, CIP Director, Office of Human Research Administration; Harvard T.H. Chan School of Public Health & Harvard Faculty of Medicine; 90 Smith Street, Room 338, Boston, MA 02120; Phone: 617–432-2153; Fax: 617–432-2165; Email: cuhs@harvard.edu.

## Abbreviations

COVID-19: Novel Coronavirus (SARS-CoV-19)
EA: Enumeration area
EVD: Ebola Virus Disease
HSCL-25: Hopkins Symptom Checklist-25
PTSD: Post-traumatic stress disorder
