# Supplementary material for: Fear, distress, and perceived risk shape stigma toward Ebola survivors: a prospective longitudinal study
Source: BMC Public Health. 2021 Nov 11;21:2066. doi: 10.1186/s12889-021-12146-0 (PMC8581958; doi:10.1186/s12889-021-12146-0)
Supplement: Supplementary file 1 — Additional file 1. [file 12889_2021_12146_MOESM1_ESM.docx]

Table S1

*EVD-related stigma scale*

| **Please tell me if you agree or disagree with the statement.**  *Duya tel mi if yu gri or nor gri wit den tin ya.* | |  |
| --- | --- | --- |
| 1. | **I would buy fresh vegetables from a shopkeeper who survived Ebola and has a certificate from a Government Health Facility stating he/she is now Ebola-free.**  *Ah go bai vegetabul to porsin way get shop way bin get ebola govement ospital don gi am pepa say e don wel?* |  |
| 2. | **If a member of my family became ill with Ebola I would want it to remain secret.**  *If wan pan mi fambul get ebola ah go wan for kip am sikrit* |  |
| 3. | **I would welcome someone back into my community neighborhood after s/he has recovered from Ebola.**  *Ah go gri for leh posin way bin get Ebola kan tap bak nay mi komuniti or mi aria way e don wel?* |  |
| 4. | **People who have had Ebola are dirty.**  *Pipul dem way bin don get ebola den dorti.* |  |
| 5. | **People who have had Ebola are cursed.**  *Pipul dem way bin get ebola den get sweh* |  |
| 6. | **People who have had Ebola should be ashamed.**  *Pipul dem way bin don get ebola for day shem.* |  |
| 7. | **People who have had Ebola should be allowed to work with children.**  *Pipul dem way bin don get ebola, for gri for leh den wok wit pikin dem.* |  |
| 8. | **People who have had Ebola must have done something wrong and deserve to be punished.**  *Pipul dem wae bin don get ebola go don du bad en den fit for sorfa.* |  |
| 9. | **People who have had Ebola should be isolated.**  *Pipul dem wae bin don get ebola den nor for miks wit oda pipul dem.* |  |
| 10. | **I want to be friends with someone who has had Ebola.**  *Ar go gri for mek padi wit porsin way bin don get Ebola.* |  |
| 11. | **People who have had Ebola should be allowed to work.**  *Pipul dem way bin don get Ebola den for gri leh den wok.* |  |
